# Lung Myofibroblasts Are Characterized by Down-Regulated Cyclooxygenase-2 and Its Main Metabolite, Prostaglandin E2

**DOI:** 10.1371/journal.pone.0065445

**Published:** 2013-06-03

**Authors:** Marta Gabasa, Dolores Royo, Maria Molina-Molina, Jordi Roca-Ferrer, Laura Pujols, Cesar Picado, Antoni Xaubet, Javier Pereda

**Affiliations:** 1 Institut d’Investigacions Biomèdiques August Pi i Sunyer (IDIBAPS), Barcelona. Spain; 2 Centro de Investigaciones Biomédicas en Red de Enfermedades Respiratorias (CIBERES), Spain; 3 Servei de Pneumologia, Grup de Recerca Pneumològic, Institut d’Investigacions Biomèdiques de Bellvitge (IDIBELL), Hospital Universitari de Bellvitge, Hospitalet de Llobregat, Barcelona, Spain; 4 Servei de Pneumologia I Al·lèrgia Respiratòria. Institut Clínic Del Tórax, Hospital Clínic, Universitat de Barcelona, Spain; 5 Departament de Fisiologia, Facultat de Farmàcia, Universitat de València, Valencia. Spain; University of Pittsburgh, United States of America

## Abstract

**Background:**

Prostaglandin E2 (PGE_2_), the main metabolite of cyclooxygenase (COX), is a well-known anti-fibrotic agent. Moreover, myofibroblasts expressing α-smooth muscle actin (α-SMA), fibroblast expansion and epithelial-mesenchymal transition (EMT) are critical to the pathogenesis of idiopathic pulmonary fibrosis (IPF). Our aim was to investigate the expression of COX-2 and PGE_2_ in human lung myofibroblasts and establish whether fibroblast-myofibroblast transition (FMT) and EMT are associated with COX-2 and PGE_2_ down-regulation.

**Methods:**

Fibroblasts obtained from IPF patients (n = 6) and patients undergoing spontaneous pneumothorax (control, n = 6) and alveolar epithelial cell line A549 were incubated with TGF-β1 and FMT and EMT markers were evaluated. COX-2 and α-SMA expression, PGE_2_ secretion and cell proliferation were measured after IL-1β and PGE_2_ incubation.

**Results:**

Myofibroblasts from both control and IPF fibroblast cultures stimulated with IL-1β showed no COX-2 expression. IPF fibroblasts showed increased myofibroblast population and reduced COX-2 expression in response to IL-1β. TGF-β1 increased the number of myofibroblasts in a time-dependent manner. In contrast, TGF-β1 induced slight COX-2 expression at 4 h (without increase in myofibroblasts) and 24 h, but not at 72 h. Both IPF and control cultures incubated with TGF-β1 for 72 h showed diminished COX-2 induction, PGE_2_ secretion and α-SMA expression after IL-1β addition. The latter decreased proliferation in fibroblasts but not in myofibroblasts. A549 cells incubated with TGF-β1 for 72 h showed down-regulated COX-2 expression and low basal PGE_2_ secretion in response to IL-1β. Immuno-histochemical analysis of IPF lung tissue showed no COX-2 immuno-reactivity in myofibroblast foci.

**Conclusions:**

Myofibroblasts are associated with COX-2 down-regulation and reduced PGE_2_ production, which could be crucial in IPF development and progression.

## Background

Idiopathic pulmonary fibrosis (IPF) is a progressive and fatal interstitial lung disease of uncertain etiology, characterized by the histopathological pattern of usual interstitial pneumonia. This fibrotic process involves the loss of lung architecture through increased epithelial cell apoptosis and abnormal wound healing, followed by the formation of fibroblast foci and excessive collagen deposition. In this context, the crucial role of myofibroblasts in tissue remodeling has been well described [1]. Myofibroblasts exhibit a contractile and collagen-secretory phenotype, characterized by the expression of α-smooth muscle actin (α-SMA). Many origins have been suggested for these cells [1]. The most important sources are probably perivascular and peribronchiolar adventitial fibroblasts, which differentiate into myofibroblasts – a process known as fibroblast-myofibroblast transition (FMT) – in a pro-fibrotic environment [1]. Moreover, evidence of the epithelial-mesenchymal transition (EMT) reveals the importance of epithelial cells as an additional source of myofibroblasts [2]. EMT involves a transition from epithelial cells to mesenchymal myofibroblast-like cells that involves a decreased expression of epithelial markers such as E-cadherin [2].

An imbalance between pro-fibrotic and anti-fibrotic mediators appears to exist in IPF. Numerous pro-fibrotic factors such as transforming growth factor (TGF)-β1 [3] and angiotensin-II [4] have been reported in IPF. In contrast, few anti-fibrotic mediators have been identified. Of the latter, prostaglandin E2 (PGE_2_) is derived from the metabolism of arachidonic acid by cyclooxygenase enzymes [5]. Experimental models of lung fibrosis show the pivotal role of this prostaglandin [6], [7]. PGE_2_ enhances epithelial-mesenchymal wound healing since it improves epithelial cell survival [8], inhibits fibroblast proliferation [9], collagen I synthesis [10], cell migration [11] and cell differentiation into myofibroblasts [12], as well as inducing fibroblast apoptosis [13]. A deficiency of PGE_2_ synthesis as a result of down-regulation of cyclooxygenase-2 (COX-2) has been described in IPF [14]–[16]. Consequently, the inability to induce COX-2 and PGE_2_ synthesis has been associated with increased fibroblast proliferation and alveolar epithelial cell apoptosis [17].

No studies to date have reported any connection between the myofibroblast phenotype and the lack of PGE_2_ in IPF. We hypothesized that the increase in myofibroblast and mesenchymal myofibroblast-like cell population observed in IPF could be related to the down-regulation of COX-2 expression and reduced PGE_2_ synthesis. Therefore, our aim was to study COX-2 regulation and PGE_2_ production in myofibroblasts and in FMT and EMT processes.

## Methods

### Population

We obtained pulmonary biopsies from patients suffering from IPF (n = 6). The diagnosis of IPF was established according to the American Thoracic Society/European Respiratory Society Consensus Statement [18]. None of the IPF patients had received corticosteroids or other immunosuppressant therapy at the time of sample collection. As for the control group, we obtained lung tissue from subjects with no history of pulmonary disease who were undergoing surgical treatment for spontaneous pneumothorax (n = 6). No histopathological evidence of disease was found in these tissue samples. Written informed consent was obtained from all patients according to institutional guidelines, and the study was approved by the Ethics Committee of the Hospital Clínic, Barcelona.

### Cell Culture and Treatments

Primary fibroblasts were isolated from the lung biopsies, using previously described methods [19]. Briefly, 1-sq.-mm fragments of tissue were incubated under sterile conditions in Dulbecco's modified Eagle's media (DMEM) (Lonza, Verviers, Belgium) supplemented with 10% fetal bovine serum (FBS), 100 IU/ml penicillin, 100 µg/ml streptomycin (Invitrogen, Carlsbad, California, USA) and 2 µg/ml amphotericin B (Sigma, St Louis, MO, USA). Cultures were kept in a 5% CO_2_ humidified atmosphere at 37°C. Fibroblasts were grown to subconfluence and subcultured by 0.05% trypsin-0.02% EDTA treatment. Fibroblasts were studied at passage 5 to 6. Culture characterization was performed by immunofluorescence for vimentin (Invitrogen) and pan-cytokeratin (C2562, Sigma), as indicated elsewhere [19]. Fibroblast cultures stained positive for vimentin and negative for cytokeratins, demonstrating the absence of epithelial cells in these cultures.

The A549 human lung adenocarcinoma epithelial cell line (American Type Culture Collection, Rockville, MD) was cultured in RPMI 1640 media (Lonza) with 1 mM L-Glutamine (Lonza) and supplemented as mentioned above.

In all the experiments, the medium was removed when the cell cultures reached 80% confluence and the cells were incubated in serum-free medium for 24 h. Three experimental models were then performed.

In the first set of experiments, fibroblasts were treated with or without IL-1β (10 ng/ml; R&D systems, Minneapolis, USA), a well-known inducer of COX-2 expression [15], for 4 and 24 h and COX-2 and α-SMA expression were measured.

In a second set of experiments, we studied the effect of TGF-β1 (5 ng/ml, R&D systems) in control and IPF cultures for 4, 24 and 72 h. We measured COX-2, α-SMA and collagen Iα1 expression. With these experiments, we established an *in vitro* model of FMT and EMT by incubating fibroblasts and A549 cells, respectively, with TGF-β1 for 72 h. EMT was also characterized by the expression of E-cadherin and immunofluorescence.

In a third set of experiments on cells treated with TGF-β1 for 72 h, we studied the effect of IL-1β (10 ng/ml for fibroblasts and 1 ng/ml for A549 cells) and PGE_2_ (5 ng/ml, Cayman chemicals, Michigan, USA) for an additional 24 h. The reasons for the use of a different concentration of IL-1β are firstly based on the literature [19], [20] and secondly based on our experience. We obtained similar PGE_2_ levels in both cells using those concentrations, allowing the comparisons between cell types. Additional TGF-β1 for 4 h and 24 h and the selective COX-2 inhibitor Celecoxib (10 µM, Sigma) were also tested. In this set of experiments TGF-β1 was removed at 72 h and fresh culture medium was added with stimuli. We measured COX-2, COX-1, α-SMA, PGE_2_ secretion and proliferation**.** We also compared these experiments with an early treatment of TGF- β1 at 4 h. The experiments were carried out on 6 tissue samples for each group, unless otherwise indicated. The experiments on A549 cells were performed in quadruplicate, at the very least.

### Western Blot

After treatment, cells plated on 90 cm^2^-diameter culture dishes were washed twice with ice-cold phosphate buffer saline (PBS). Cells were lysated directly with 200 µl of RIPA buffer [TrisHCl 50 mM, NaCl 150 mM (pH 7,4), 1% NP40 Igepal, 1% Triton X-100, 0.1% SDS and 5 µl/ml of a protease inhibitor cocktail (Sigma). The lysate was maintained for 20 min on ice and was then collected and frozen at −80°C. Samples were thawed, sonicated twice for 15 s in a Sonifier (Branson, Danbury, CT, USA) and immediately centrifuged at 20,000 g for 10 min at 4°C for protein measurement. 12.5–30 µg of protein from fibroblasts and A549 cells were denaturalized in a thermocycler (70°C, 10 min) in loading buffer (NuPAGE LDS sample buffer), loaded on 7% Tris-Acetate gels and run (125 V, 90 min) in a Novex XCell ΙΙ Mini-Cell (Invitrogen, San Diego, CA, USA). The proteins were then transferred to a nitrocellulose membrane and non-specific binding sites were blocked using blocking buffer (5% non-fat dry milk, 0.1% Tween 20 in PBS). Membranes were incubated overnight at 4°C with 1∶1.000 dilution in blocking buffer of the primary antibodies against COX-2 (Santa Cruz Biotechnology, Santa Cruz, CA, USA), COX-1 (Santa Cruz Biotechnology), α-SMA (Sigma), E-cadherin (BD Biosciences, Bedford, MA, USA) or p44/42 MAPK (Erk1/2) (Cell Signalling Technology) and 1∶10.000 dilution for β-actin (Sigma). Blots were then washed in 0.5% Tween 20 in PBS (T-PBS) and subsequently incubated for 2 h at room temperature with an appropriate horseradish peroxidase-labeled secondary antibody diluted 1∶3,000 in blocking buffer. After washing with T-PBS, blots were incubated with an enhanced chemiluminiscent substrate (Supersignal West Pico Chemiluminescent Substrate, Rockford, IL, USA). Quantification of protein expression was carried out using a CCD Camera System LAS 3000 (Fujifilm, Tokyo, Japan) and densitometry was performed by Image gauge v.4.0 software. For the fibroblast Western blots, results were presented as a ratio to the expression of β-actin. To check for equal loading in A549 cells, results were expressed as a ratio of band density to total p44/42 MAPK (Erk1/2) since the levels of β-actin underwent changes after TGF- β1 treatment (data not shown).

### Immunofluorescence

Cells grown in 4-well CultureSlides® were fixed with cold 4% paraformaldehyde for 15 min and then permeabilized with 0.5% Triton X-100 for 30 min. Once blocked with 1% bovine serum albumin-PBS for 1 h, primary antibodies against α-SMA (Sigma, 1∶500) or COX-2 (Santa Cruz Biotechnology, 1∶500) were added for 1 h at 37°C. Secondary antibodies or Phalloidin-TRITC (Sigma) were also incubated for 1 h. Cells were counterstained with DAPI (1∶10.000) and were mounted with Prolong® Gold antifade reagent (Invitrogen, Molecular Probes).

For double-staining experiments, COX-2 antibody and its corresponding secondary antibody were first incubated for 45 min each, and thereafter α-SMA and its secondary antibody were incubated for another 45 min each. This order was established empirically in a set of experiments and demonstrated very low cross-reactivity between antibodies. Phalloidin also showed no cross-reactivity with COX-2 primary and secondary antibodies and was incubated together with COX-2 secondary antibody. Epifluorescence microscopy (Leica Microsystems, Germany) was used to analyze preparations at ×200 or ×400 magnification. Positive cells for α-SMA and COX-2 were counted in 12 random fields.

### Immunohistochemistry

Histological sections were obtained from paraffin-embedded biopsies of control (n = 5) and IPF patients (n = 5). Paraffin was removed and sections were heated at 80°C for 40 min in citrate buffer (0.01 M, pH 6) for the antigen retrieval process. Then, samples were blocked in PBS, 0.05% v/v Triton X-100, 3% goat serum (Sigma) for 30 min at room temperature. Primary antibodies against COX-2 (Santa Cruz Biotechnology) and α-SMA (Sigma) diluted 1∶1000 in blocking solution were incubated overnight at 4°C. The next steps were performed using EnVision™ Detection Systems Peroxidase/DAB, Rabbit/Mouse kit (Dako, Denmark), according to the manufacturer’s instructions. Nuclei were contrasted with Gill I Hematoxylin for 1 min. Sections were mounted using Glycergel® Mounting Medium (Dako).

### Analysis of DNA Replication (Click-iT®)

A commercial kit was used, based on the incorporation of EdU (5-ethynyl-2′-deoxyuridine), a modified nucleoside that is incorporated during DNA synthesis (Click-iT®, Invitrogen). After treatment, cells grown in 90-sq.-cm dishes were incubated with 10 µM EdU for 2 h before being harvested them for flow cytometry measurements (Click-iT® EdU Alexa Fluor® 488 Flow Cytometry Assay Kit, Invitrogen). The next steps were performed in accordance with the manufacturer's instructions. The technique was validated with a set of experiments incubating cells with increasing concentrations of serum. Results were presented as percentage of cells incorporating the modified nucleoside EdU. Negative control was also measured to establish positive criteria.

### Collagen Expression

Total RNA was isolated with the RNeasy mini kit (Qiagen, Mississauga, ON, CA) according to the instructions of the manufacturer. One µg of RNA was reverse-transcribed into cDNA with the High Capacity cDNA Reverse Transcriptase kit with RNase Inhibitor (Applied Biosystems, Life Technologies, Wellesley, CA) using random primers, optimized RT Buffer, dNTP’s and MultiScribe™ MuLV reverse transcriptase. cDNA was subjected to amplification by real-time PCR (7900 HT Fast Real-Time PCR System, Applied Biosystems) using TaqMan® Gene Expression Assays (Applied Biosystems) for collagen type I, alpha 1 (COLIA1; Assay Id: Hs00164004_m1) and RNA polymerase II, polypeptide A (RPII; Assay Id: Hs00172187_m1) as a constitutive endogenous gene. Data were calculated using the ΔΔCt method and the results expressed as fold-change related to control samples at time 0 h.

### Sircol Collagen Assay

Total soluble collagen in cell culture supernatants was quantified using the Sircol collagen assay (Biocolor, Belfast, UK). Briefly, 500 µl of fibroblast culture supernatant were collected and concentrated overnight using the kit’s concentration reagent. 1 ml of Sirius red dye was added and incubated with gentle rotation for 30 min at room temperature. After centrifugation at 12,000 g for 10 min, the collagen-dye pellet was washed with 750 µL of cold Acid-Salt Wash Reagent and centrifuged again. The pellet was re-dissolved with 250 µL of 0.5 M NaOH, and absorbance at 540 nm was measured by microplate ELISA reader. The results are presented as total µg Collagen secreted/µg total protein content.

### PGE_2_ Assay

PGE_2_ Assay was performed in cell culture medium using Prostaglandin E_2_ EIA Kit (Cayman chemicals). Supernatants were filtered through 0.22 µm filter (BD Biosciences) and stored at −80°C. The measurement was performed according to the manufacturer’s instructions. Results are presented as total pg PGE_2_ secreted/µg total protein content.

### Statistical Data Analysis

All statistical analyses were performed with SPSS 14.0 (SPSS, Chicago, IL USA). Data are expressed as median and 25th to 75th percentile. Immunofluorescence cell count results are presented in tables as mean values ± SD. The non-parametric statistical Mann-Whitney U-test was used for control and IPF group comparisons and the Wilcoxon test was used for paired comparisons. Parametric statistical analyses for A549 cells experiments were performed with ANOVA followed by Dunnett’s multiple comparison post hoc tests. Differences were considered to be significant if *p-*values were below 0.05.

## Results

### Expression of COX-2 and α-SMA in Control and IPF Fibroblasts

**Figure 1:**
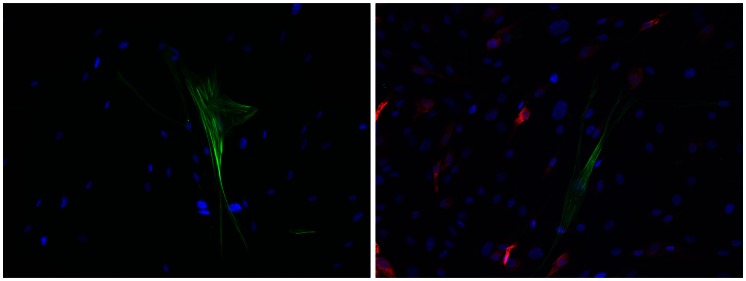
Co-immunofluorescence of COX-2 (red) and α-SMA (green) of a representative control fibroblast culture. In basal conditions (left), it shows no COX-2 expression and isolated α-SMA-positive cells, and after IL-1β treatment for 24 h (right), it shows high COX-2 expression and a lack of COX-2 immunoreactivity in α-SMA-positive cells. Nuclei were stained with DAPI (blue). (Original magnification X200).

IPF cultures showed increased population of myofibroblasts (α-SMA positive cells) basally and reduced population of COX-2 positive cells in response to IL-1β (10 ng/ml for 24 h) compared with control cells, as observed by immunofluorescence (Table 1). As described elsewhere [15], [16], western blot showed a diminished induction of COX-2 expression in response to IL-1β stimulation in IPF fibroblasts compared to control cells (Fig. S1). A significant decrease in α-SMA positive cells was found after 24 h of IL-1β treatment in both groups. Interestingly, α-SMA positive cells showed little or no expression of COX-2 in response to IL-1β (Table 1, Fig. 1). This finding demonstrates that spontaneous myofibroblasts present in culture are characterized by diminished COX-2 expression in response to IL-1β.

**Table 1:** Quantification of immunofluorescence of COX-2, α-SMA and COX-2+ α-SMA.

Treatment (24 h)	Determinations	Control Group	IPF Group
None	COX-2	ND	ND
	α-SMA	2.8±1.6%	9.4±3.7%**
IL-1β (10 ng/ml)	COX-2	48.05±11.09%	31.7±5.7%*
	α-SMA	1.6±2.7% †	5.1±2.8%* †
	COX-2 & α-SMA	0.01±0.10%	0.17±0.11%

Quantification of immunofluorescence of COX-2, α-SMA and COX-2+ α-SMA. Control and IPF fibroblasts were incubated for 24 h in the presence or absence of IL-1β (10 ng/ml). Results are presented as mean ± SD of the percentage of total cells. ND, not detected.

* P<0.05 and **P<0.01 compared to control group,

† P<0.05 compared to the respective untreated cells.

### Induction of Fibroblast-myofibroblast Transition by TGF-β1

**Figure 2:**
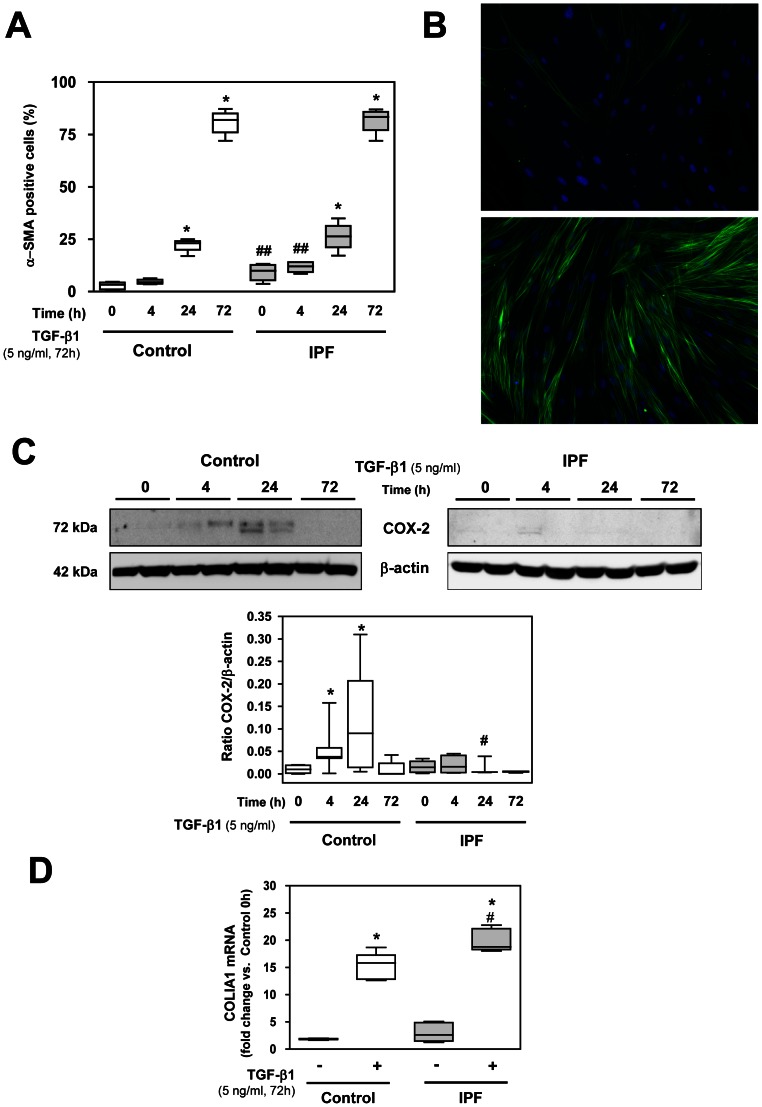
Induction of fibroblast-myofibroblast transition (FMT) in control and IPF fibroblasts by TGF-β1 incubation. (A) Quantification of α-SMA positive cells (myofibroblasts) by immunofluorescence. Results are presented as percentage of total cells. (B) Representative immunofluorescence showing the effect of 5 ng/ml TGF-β1 for 72 h in control fibroblasts. The expression of α-SMA is shown in green and the nuclei were stained with DAPI (blue) (Original magnification X200). (C) Representative Western blot of COX-2 and β-actin in two fibroblast cultures obtained from both control and IPF patients in response to TGF-β1 treatment at 4, 24 and 72 h. Densitometric analysis of COX-2 expressed as a ratio versus β-actin is also shown. N = 6 Control cells. N = 4–6 IPF cells. (D) Collagen Iα1 mRNA measured by real-time PCR with or without TGF-β1 treatment (5 ng/ml) for 72 h in control and IPF fibroblasts. *P<0.05 compared to respective untreated cells, ^#^P<0.05 and ^##^P<0.01 compared to control group.

In order to induce myofibroblasts in our cultures, we used TGF-β1 (5 ng/ml). This treatment provoked a significant increase in myofibroblasts (α-SMA-positive cells) in a time-dependent manner, reaching statistically significant values at 24 and 72 h in both cultures (Fig. 2A). Interestingly, the TGF-β1 treatment induced a similar amount of myofibroblasts in the control and IPF cell cultures at both points. Consequently, the differences between control and IPF in the number of myofibroblasts in non-treated cells were lost. Representative images of TGF-β1 induction of FMT are presented (Fig. 2B).

In the control fibroblasts, TGF-β1 stimulated COX-2 expression slightly at 4 h and more markedly at 24 h but no COX-2 expression was observed at 72 h. In IPF treated fibroblasts, no COX-2 expression was observed after TGF-β1 treatment (Fig. 2C). Consequently, in control fibroblasts, we observed COX-2 expression at 4 h induced by TGF-β1 without any statistical changes in the number of myofibroblasts and the opposite situation in long-term treatment with TGF-β1 (72 h), which is characterized by a myofibroblast-enriched population with no expression of COX-2. RT-PCR showed a marked up-regulation of collagen Iα1 synthesis in fibroblasts after TGF-β1 treatment, a characteristic feature of the myofibroblast phenotype. This increase was significantly higher in IPF samples treated with TGF-β1 compared to control treated samples (Fig. 2D).

We also performed Sircol assay in order to measure the secreted collagen in IPF and Control fibroblast cultures under the same experimental conditions. The results are presented as total µg Collagen secreted/µg total protein content. Although the same pattern found in collagen mRNA expression was observed, no significant differences were found between control and IPF fibroblasts. (Control 0 h: 0.035±0.02; IPF 0 h: 0.043±0.01; Control 72 h: 0.078±0.02*; IPF 72 h: 0.084±0.03*; Control+TGF-β1 72 h: 0.122±0.04*†; IPF+TGF-β1 72 h: 0.135±0.03**††). (*p<0.05 and ** p<0.01 vs. 0 h; † p<0.05 and †† p<0.01 vs. 72 h without treatment). N = 6 each. We found Sircol analysis insufficiently sensitive to find these differences in our samples.

### Myofibroblast Phenotype from FMT Results in Down-regulation of COX-2 Expression

**Figure 3:**
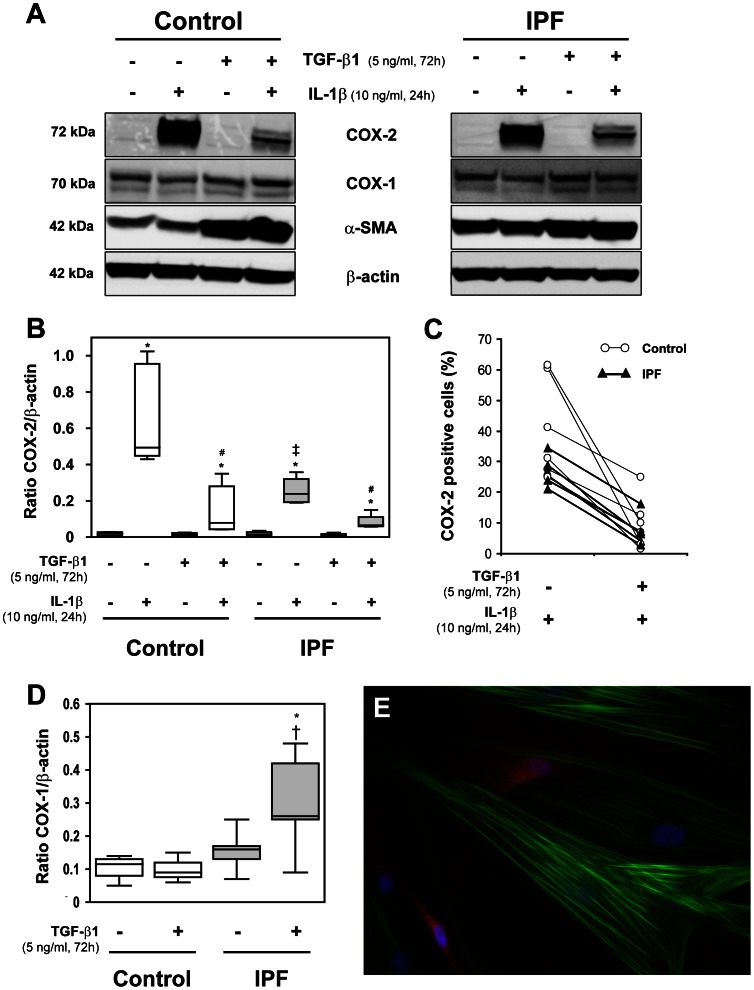
Effect of IL-1β stimulation on a myofibroblast-enriched population. (A) Protein levels in control and IPF fibroblasts of COX-2, COX-1, α-SMA and β-actin at 72 h with or without TGF-β1 treatment (5 ng/ml), and at subsequent 24 h in the presence or absence of IL-1β (10 ng/ml), measured by Western blot. (B) Densitometric analysis of COX-2 expressed as a ratio versus β-actin. (C) Quantification of COX-2 positive cells by immunofluorescence. Results are presented as percentage of total cells. (D) COX-1/β-actin ratio at 72 h in the presence or absence of TGF-β1 (5 ng/ml). *P<0.05 compared to untreated cells, ^#^P<0.05 compared to IL-1β treated cells, ^†^P<0.05 and ^‡^P<0.01 compared to control group in the same conditions. (E) Representative co-immunofluorescence image of COX-2 (red) and α-SMA (green) of a myofibroblast-enriched culture obtained from control fibroblasts stimulated with IL-1β for 24 h. Nuclei were stained with DAPI (blue). Note that myofibroblasts do not express COX-2 in response to IL-1β and the different shape of the COX-2 positive cells (Original magnification X400).

We studied COX-2 and α-SMA expression in the myofibroblast-enriched cultures obtained as described in Figure 2. Both groups presented a marked increase in α-SMA expression when treated with TGF-β1 (Fig. 3A). The TGF-β1-treated cells showed a dramatic decrease in COX-2 expression in response to IL-1β (Fig. 3A, B).

Consequently, an increase in α-SMA positive cells was associated with a decrease in COX-2 positive cells induced by IL-1β (Fig. 3C). Cells positive for COX-2 were almost absent in the TGF-β1-treated cultures. The cell counting of co-immunofluorescence is summarized in Table 2. As expected, an extremely low number of α-SMA positive cells were also positive for COX-2 after IL-1β treatment.

**Table 2:** Quantification of immunofluorescence of COX-2, α-SMA and COX-2+ α-SMA.

Treatment		Determinations	Control Group	IPF Group
None (72 h)	None (24 h)	COX-2	ND	ND
		α-SMA	2.9±9%	9.2±2.9% **
	IL-1β (10 ng/ml, 24 h)	COX-2	41.2±16.3% †	26.7±5.2% * †
		α-SMA	1.7±2.5% †	6.05±4.1% * †
		COX-2 & α-SMA	0.02±0.07%	0.14±0.09%
TGF-β1 (5 ng/ml, 72 h)	None (24 h)	COX-2	ND	ND
		α-SMA	82.3±5.3% ‡	79.9±8.9% ‡
	IL-1β (10 ng/ml, 24 h)	COX-2	9.9±8.5% ‡	7.4±5.3% ‡
		α-SMA	70.7±11.9% ‡ §	72.4±11.04% ‡ §
		COX-2 & α-SMA	0.4±0.5%	0.3±0.07%

Quantification of immunofluorescence of COX-2, α-SMA and COX-2+ α-SMA. Control and IPF fibroblasts were incubated for 72 h in the presence or absence of TGF-β1, followed by further incubation in the absence or presence of IL-1β (10 ng/ml) for 24 h. Results are presented as mean ± SD of the percentage of total cells. ND, not detected,

* P<0.05 and ** P<0.01 compared to control group in the same conditions,

† P<0.05 compared to the respective untreated cells,

‡ P<0.05 compared to non-treated TGF-β1 cells.

§ P<0.05 compared to percentage of α-SMA in TGF-β1 treated cells in the absence of IL-1β.

COX-1 expression increased after TGF-β1 treatment only in IPF fibroblasts (Fig. 3D), whereas it remained unmodified by IL-1β in both groups (densitometry not shown).

Immunofluorescence revealed that cells positive for COX-2 presented a thin fibroblast-like shape and α-SMA negativity. Consequently, myofibroblasts (α-SMA positive cells) showed no positive staining for COX-2 (Fig. 3E, Table 2).

Re-stimulation of myofibroblasts with TGF-β1 for additional 4 and 24 h produced no COX-2 expression, as expected (Fig. S3).

### Induction of Epithelial-mesenchymal Transition by TGF-β1 Results in Down-regulation of COX-2 Expression

**Figure 4:**
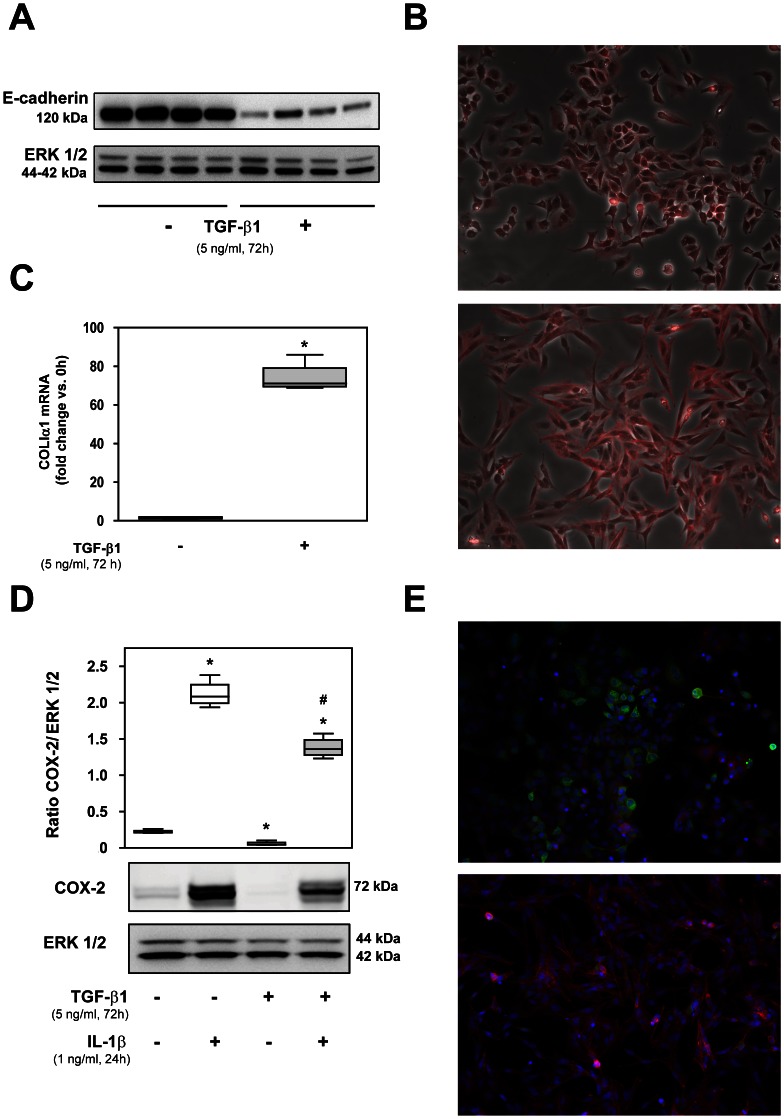
Induction of epithelial-mesenchymal transition (EMT) in A549 cells by TGF-β1 incubation and effect of IL-1β in transformed cells. Effect of 5 ng/ml TGF-β1 for 72 h in A549 cell culture. (A) Representative Western blot of four replicates of E-cadherin and ERK 1/2. (B) Representative immunofluorescence showing the expression of F-actin (stained with phalloidin, red) and bright field (Original magnification X400). (C) Collagen Iα1 mRNA measured by real-time PCR. Effect of IL-1β for 24 h (1 ng/ml) in A549 cells after incubation with 5 ng/ml TGF-β1 (72 h). (D) Representative Western blot and densitometric analysis of COX-2 expression versus ERK 1/2. *P<0.05 compared to untreated cells, ^#^P<0.01 compared to IL-1β-treated cells. (E) Immunofluorescence of COX-2 (green) and F-actin (stained with phalloidin, red) in cells cultured in absence (upper) or presence (lower) of TGF-β1. In both images, cells were stimulated with IL-1β (1 ng/ml). Nuclei were stained with DAPI (blue). Note the almost complete absence of COX-2 staining in TGF-β1-treated cells (original magnification X400).

We studied EMT by treating A549 cells with TGF-β1 (5 ng/ml, 72 h), as described above. Epithelial-specific marker E-cadherin dramatically decreased in treated A549 cells (Fig. 4A). Immunofluorescence using phalloidin (F-actin marker) showed a strong red staining in A549 cells incubated with TGF-β1, revealing cytoskeleton remodeling and a morphological change into a myofibroblast-like phenotype (Fig. 4B). RT-PCR showed a marked up-regulation of collagen Iα1 synthesis in A549 cells after TGF-β1 treatment (Fig. 4C). Weak expression of α-SMA and no expression of COX-1 in A549 were found, either basally or after stimulation with TGF-β1 (results not shown).

Unlike fibroblasts, A549 cells expressed COX-2 in the absence of IL-1β stimulation (Fig. 4D). Interestingly, TGF-β1 treatment for 72 h decreased this basal COX-2 expression. We then observed an increase in COX-2 expression in response to IL-1β (1 ng/ml), which was reduced in TGF-β1-treated cells (Fig. 4D). This inhibition can be clearly observed by immunofluorescence (Fig. 4E).

### Myofibroblast Phenotype from FMT and EMT Implies a Decrease in PGE_2_ Synthesis

**Figure 5:**
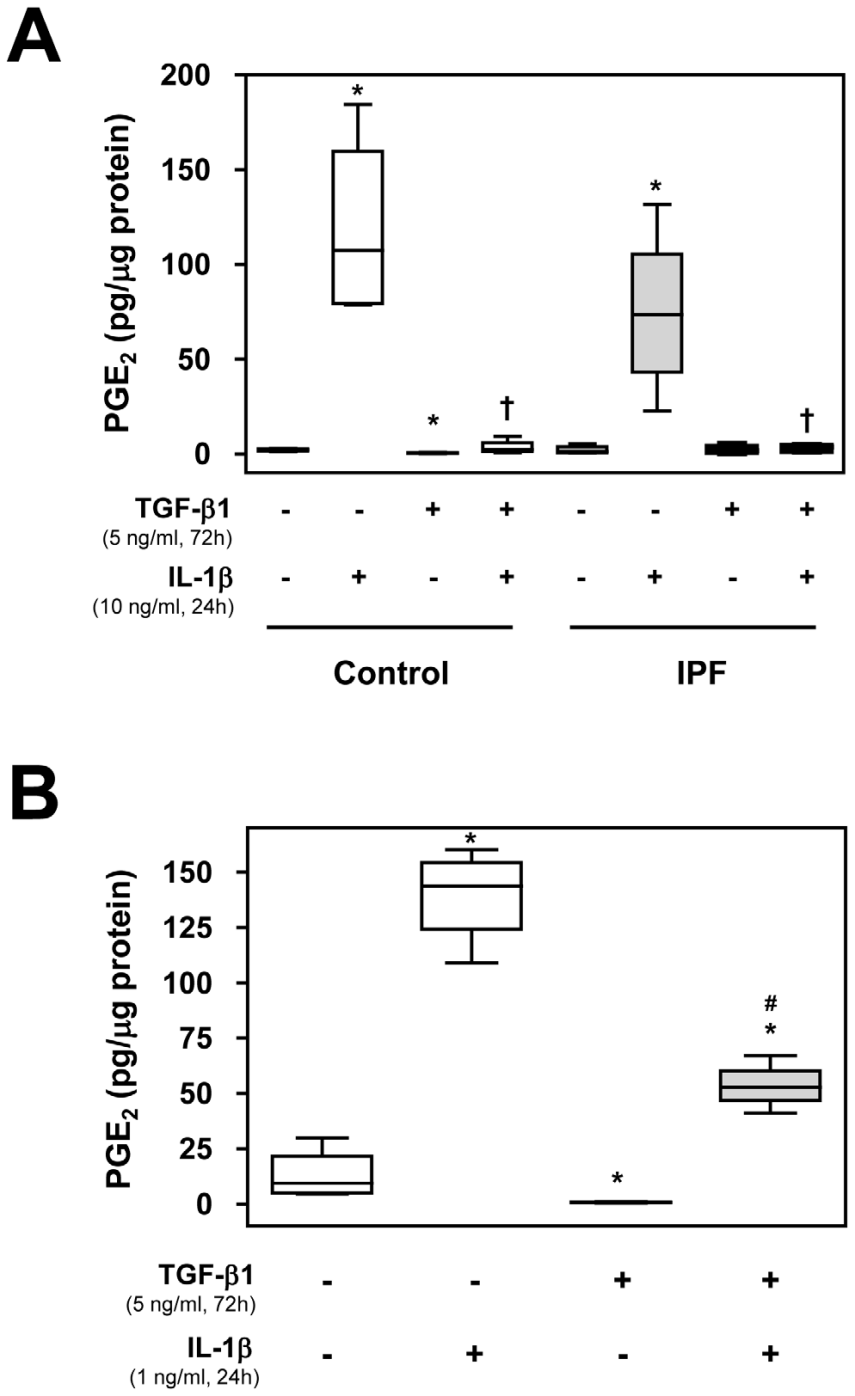
Measurement of PGE_2_ secretion after FMT or EMT induction. Control and IPF fibroblasts (A) and A549 cells (B) were incubated for 72 h in the presence or absence of TGF-β1 (5 ng/ml), followed by treatment in the presence or absence of IL-1β (10 ng/ml or 1 ng/ml, respectively) for 24 h. Supernatant was then collected and total PGE_2_ was measured using Prostaglandin E_2_ EIA Kit (Cayman). Results are expressed as PGE_2_ secreted/µg total protein content. *P<0.05 compared to untreated cells, ^†^P<0.05 and ^#^P<0.01 compared to IL-1β-treated cells.

We examined PGE_2_ levels in culture supernatants of control, IPF fibroblasts (Fig. 5A) and A549 cells (Fig. 5B) treated with TGF-β1. IL-1β incubation increased PGE_2_ secretion in fibroblasts and A549 cells. This effect was diminished in TGF-β1 pretreated cells (Fig. 5A, B). The results correlated with COX-2 Western blot analysis and immunofluorescence. Therefore, a myofibroblast-like phenotype is associated with a notable decrease in PGE_2_ secretion.

### COX-2 Down-regulation of Myofibroblast-like Phenotype Alters Cell Proliferation

We studied the role of COX-2 and PGE_2_ in myofibroblast-enriched culture proliferation by the Click-iT® technique. Fibroblasts and A549 cells were incubated with IL-1β (10 ng/ml and 1 ng/ml respectively), PGE_2_ (5 ng/ml) and the selective COX-2 inhibitor Celecoxib (10 µM) for 24 h.

**Figure 6:**
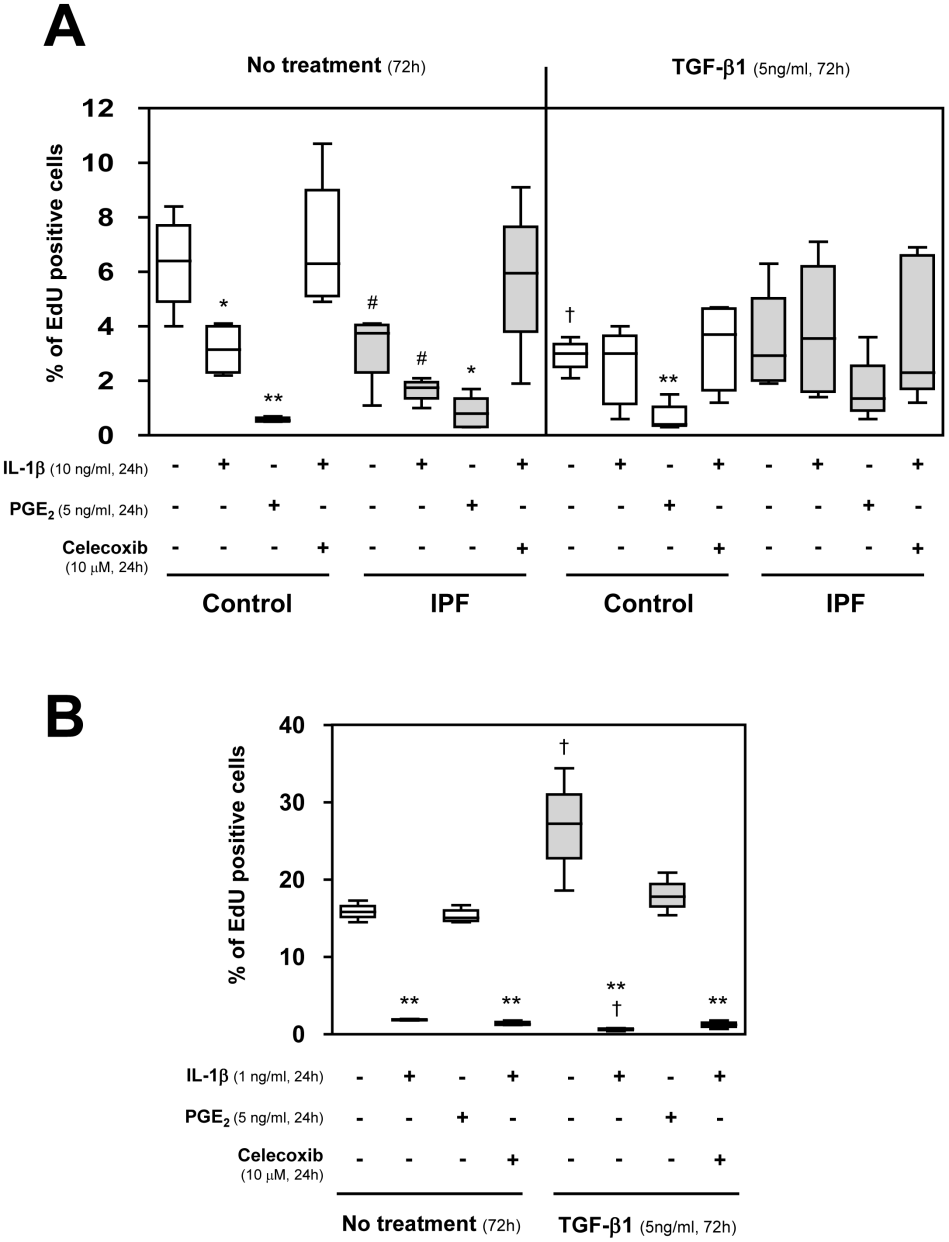
Cell proliferation measured by the analysis of DNA replication. For this purpose, control and IPF fibroblasts (A) and A549 cells (B) were incubated for 72 h in the presence or absence of TGF-β1 (5 ng/ml), followed by treatment in the presence or absence of IL-1β (10 ng/ml or 1 ng/ml, respectively), PGE_2_ (5 ng/ml) and the selective COX-2 inhibitor Celecoxib (10 µM). Cell proliferation was then analyzed by measuring the incorporation of the modified nucleoside EdU into the DNA using the Click-iT® commercial kit. Cells were incubated with 10 µM EdU for 2 h before being harvested for flow cytometry measurements. Results are expressed as the percentage of EdU positive cells. *P<0.05 and **P<0.01 compared to respective untreated cells, ^#^P<0.05 compared to control group in the same conditions, ^†^P<0.05 compared to non-treated TGF-β1 cells in the same conditions.

In fibroblast control cells, IL-1β treatment provoked a significant decrease in proliferation (Fig. 6A). This decrease appears to be mediated by COX-2 since PGE_2_ completely abrogated cell proliferation and the addition of Celecoxib reversed the IL-1β effect. IPF fibroblasts presented a diminished proliferation compared with control fibroblasts, but similar effects were observed after incubation with IL-1β, PGE_2_ and Celecoxib. Moreover, TGF-β1 treatment induced a decrease in control fibroblast proliferation but no differences were observed in IPF fibroblast cultures. Interestingly, the inhibitory effect on proliferation associated with IL-1β exposure was annulled in TGF-β1 treated cells (Fig. 6A).

In A549 cells, incubation with IL-1β significantly reduced cell proliferation in both TGF-β1-treated and non-TGF-β1-treated cells (Fig. 6B). This effect seems to be independent of COX-2 and PGE_2_ since Celecoxib was not able to restore control levels and PGE_2_ itself had no effect on cell proliferation. Furthermore, we observed that TGF-β1 treatment significantly increased cell proliferation.

### Myofibroblast Foci in IPF Lungs are Characterized by the Presence of α-SMA and Absence of COX-2

**Figure 7:**
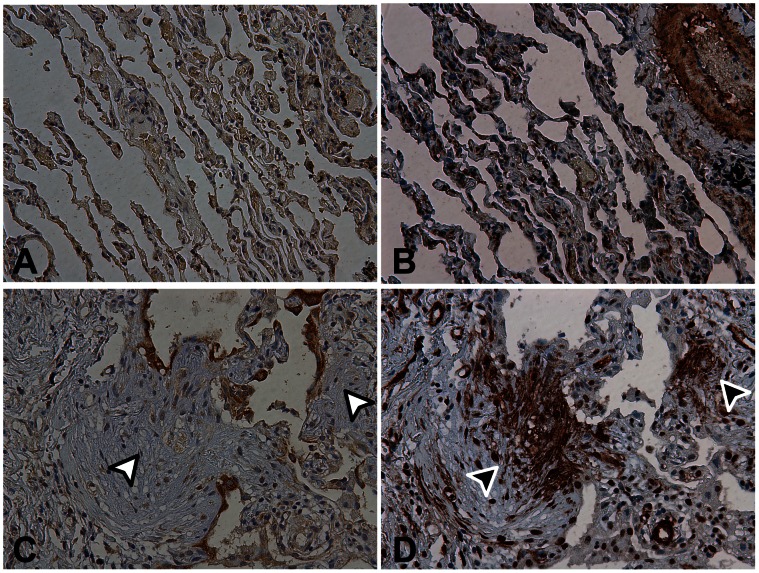
Immuno-histochemical detection of COX-2 and α-SMA in healthy lung tissue (A–B) and in idiopathic pulmonary fibrosis tissue (C–D). Immuno-localizations of COX-2 (A–C) and α-SMA (B–D) are shown. The image shows slight basal expression of COX-2 and isolated α-SMA staining related to α-SMA-positive cells in control tissue. In contrast, increased α-SMA staining (black arrows) and absence of COX-2 expression (white arrows) are observed in fibroblastic foci. Metaplastic epithelium presents widespread expression of COX-2 (Original magnification X200).

The localization of α-SMA and COX-2 was examined by immunohistochemistry in IPF and control lung tissue. Epithelial cells from control tissue were slightly stained for COX-2 (Fig. 7A) and staining of α-SMA was present in airway smooth muscle cells (Fig. 7B). Moreover, in IPF tissue α-SMA was strongly localized in fibroblast/myofibroblast foci (Black arrows, Fig. 7D). Interestingly, metaplastic epithelial cells presented strong COX-2 staining but fibroblast/myofibroblast foci showed no COX-2 expression (White arrows, Fig. 7C). Immuno-histochemical staining for α-SMA and COX-2 was negative in histological slides incubated without the primary antibody.

## Discussion

IPF is characterized by increased expression of TGF-β1, increased myofibroblast numbers and reduced production of PGE_2_ resulting from a limited induction of COX-2. We postulated that the three findings are somehow related. Consequently, we hypothesized that myofibroblasts are characterized by an impaired COX-2 expression and reduced PGE_2_ secretion.

We first confirmed previous studies reporting that IPF fibroblasts cultures are characterized by reduced COX-2 expression [15] but increased α-SMA [21], [22] compared with control fibroblast cultures. Since COX-2 was not detected at baseline, stimulation with IL-1β was performed. This first confirmation suggests that our cell lines behave as published by other groups. We must point out that the use of lung fibroblasts obtained from patients with spontaneous pneumothorax as subrogate of “normal” (control) lung has some limitations. Although histological analysis of control tissue was normal in our patients, previous studies have reported that fibroblasts obtained from surgical resections during pleurodesis for spontaneous pneumothorax, may proceed from areas with pathological abnormalities, including inflammatory and fibrotic reactions [23]. To the best of our knowledge, however, there are not other better options to obtain samples from healthy or minimally affected lung tissue.

We secondly analyzed the relationship between COX-2 expression and the myofibroblast phenotype using an immunofluorescence technique. Interestingly, myofibroblasts stained negative for COX-2 after IL-1β stimulation, suggesting that the myofibroblast phenotype is characterized by an down-regulated COX-2 expression. Cells expressing COX-2 were thinner, smaller and sharper than myofibroblasts. IL-1β treatment also decreased the number of myofibroblasts, thereby confirming this cytokine as a potent inhibitor of FMT, as has been described [12]. We could not find any morphological difference between fibroblasts expressing COX-2 and those not expressing it.

All in all, these findings suggest that myofibroblast transformation eventually results in the down-regulation of COX-2. The presence of more myofibroblast in fibroblast cultures and in the lung in IPF may account for the reported low induction of COX-2 in IPF tissues. However, the small difference between the percentages of myofibroblasts in IPF and control lung fibroblast cultures – together with the observation that COX-2-expressing fibroblasts were apparently indistinguishable from non-COX-2-expressing fibroblasts – suggests that the mechanisms involved in the down-regulation of COX-2 may also be present in IPF fibroblasts (α-SMA negative cells).

To gain insight into the relationship between myofibroblast phenotype and COX-2 regulation, the myofibroblast numbers were increased by inducing the transition of fibroblast into myofibroblast with TGF-β1. As previously described, we observed that TGF-β1 myofibroblast induction was time and dose-dependent [12] and was accompanied by a strong increase in collagen Iα1 expression [1], with greater expression of collagen Iα1 in IPF cultures than in control cultures. In contrast, the Sircol method was not sensitive enough to find any significant differences between control and IPF cells in our samples. The percentage of myofibroblasts markedly increased in cultures, to the same extent as in the IPF and control fibroblasts.

Previous reports have demonstrated that TGF-β1 increases COX-2 expression and PGE_2_ synthesis in fibroblasts over short periods (2–24 h) [24]–[26]. These reports agree with our results, since we observed increased COX-2 induced by TGF-β1 at 4 and 24 h in control fibroblasts. The number of myofibroblasts remained low at those time points. At longer times (72 h), the high amount of myofibroblasts resulted in reduced COX-2 expression. Moreover, after we treated control cells with IL-1β from that time point for additional 4 or 24 h, COX-2 expression was reduced dramatically. In contrast, we could not observe induction of COX-2 expression in IPF cultures after TGF-β1 treatment, even after short periods. We performed experiments at 4 h with and without TGF-β1 and with and without IL-1β and we observed that, together, both treatments enhanced COX-2 expression, as described elsewhere (see Fig. S2) [27]. We cannot rule out therefore that FMT is characterized by an initial phase with a slight up-regulation of COX-2. Another possibility is that incubation with TGF-β1 produces an increase in COX-2 expression, independent of FMT. Future experiments (e.g. the use of other FMT inducers) would help us to clarify this matter and to accurately determine the stage at which myofibroblasts finally down-regulate COX-2.

Consequently, the number of cells expressing COX-2 after stimulation with IL-1β after 72 h of TGF-β1 decreases in parallel with the increase in the number of fibroblasts transformed into myofibroblasts. In addition, re-incubation with TGF-β1 for an additional 4 and 24 h only induced COX-2 if the culture was not enriched with myofibroblasts (see Fig. S3), discarding a possible artifact of rapid expression/degradation of the enzyme. However, in those long-term studies, the level of COX-2 induction in response to IL-1β was not as homogeneous as in short-term studies and variability was found, mainly in control cells and not related to technique employed. Interestingly, patients with the highest levels of COX-2 after IL-1β stimulation in long-term studies showed the lowest numbers of α-SMA positive cells before IL-1β treatment and vice versa. The origin of the variability in terms of α-SMA or in terms of COX-2 expression is unknown but both parameters are clearly related as we have shown. Since TGF-β1 increases dramatically α-SMA positive cells in all cell lines studied, reduced COX-2 expression after TGF-β1 treatment is consistent in all the patients studied.

With long-term studies we confirmed our previous observations linking down-regulation of COX-2 with the myofibroblast phenotype. We also investigated the secretion of PGE_2_ in myofibroblast-enriched cultures. PGE_2_ secretion induced by IL-1β in TGF-β1-treated fibroblasts was strongly reduced in both the control and IPF patients, as a result of the inhibition of COX-2 caused by long-term exposure to TGF-β1.

The incubation of TGF-β1-treated cells with IL-1β provoked a reduction in α-SMA positive cells, suggesting that IL-1β not only inhibits myofibroblast cell transition but also promotes the disappearance of myofibroblasts in culture. Interestingly, COX-1 expression only increased significantly after TGF-β1 treatment in fibroblasts obtained from IPF patients. Since the reduction of COX-2 in IPF fibroblast cultures is notable, we are tempted to speculate that these cells may respond to TGF-β1 treatment by increasing COX-1 expression to maintain basal COX metabolism.

We studied A549 cells, which are widely used as a model of EMT [28], [29]. However, the tumour origin of the cell line limits the extrapolation of the results to primary epithelial pulmonary cells. These cells are useful for studying COX-2 expression since they do not express COX-1 and show high basal COX-2 expression [30]. In A549 cells, TGF-β1 treatment decreased basal COX-2 expression and provoked a diminished COX-2 induction after IL-1β stimulation. Accordingly, PGE_2_ levels paralleled COX-2 expression. Therefore, the EMT process leading the myofibroblast-like phenotype could also contribute to the down-regulation of COX-2 and PGE_2_ observed in IPF.

We also examined the interrelationship between TGF-β1, IL-1β, COX-2 and PGE_2_ in proliferation once the cells were transformed into myofibroblasts. Interestingly, treatment with IL-1β decreased proliferation in control fibroblasts. This effect may be mediated by COX-2 activation, since the selective COX-2 inhibitor Celecoxib completely restored cell proliferation. In contrast, the anti-proliferative effect of IL-1β was lost in myofibroblasts from both the control and the IPF patients. The lack of any effect of IL-1β on myofibroblasts appears to be the result of impaired COX-2 induction. Consequently, the exogenous addition of the COX-2 metabolite PGE_2_ decreased cell proliferation not only in fibroblasts but also in myofibroblasts. Our findings agree with reports of the anti-proliferative effects of PGE_2_ in various cell types [9].

The incubation of A549 cells with TGF-β1 may inhibit epithelial cell proliferation [31]. We studied the proliferation of A549s after their transformation into myofibroblast-like phenotype by TGF-β1 treatment. In these conditions, we found increased cell proliferation in cells treated with TGF-β1 compared to untreated cells. The treatment of A549 cells with IL-1β provoked a remarkable decrease in proliferation compared with untreated cells. This effect does not seem to be mediated by COX-2 and PGE_2_, since the COX-2 inhibitor Celecoxib did not restore proliferation and PGE_2_ treatment had no inhibitory effect on cell proliferation. Our results suggest that PGE_2_ may modulate cell proliferation specifically in FMT but not in EMT, and they point to PGE_2_ as a potential therapeutic treatment for IPF, largely on account of its targeting of myofibroblasts. A recent report has demonstrated that PGE_2_ deficiency in IPF results in increased alveolar epithelial cell apoptosis and reduced sensitivity of fibroblasts to apoptosis [17]. Consequently, PGE_2_ may also regulate apoptosis differentially in fibroblasts and epithelial cells.

We also studied histological slides of control and IPF human lungs. In line with previous reports [32], we observed a slight basal COX-2 expression in epithelial cells and α-SMA staining in airway smooth muscle cells from control lungs. Little or no COX-2 staining was observed, however, in the fibroblast and myofibroblast foci of IPF patients. Moreover, we observed, in keeping with Lappi-Blanco [33], increased COX-2 staining in epithelial metaplastic tissue in IPF. Therefore, cells that maintain the epithelial phenotype have high COX-2 expression while fibroblast/myofibroblast areas do not express the enzyme. Clearly, our model of A549 cells does not mirror metaplastic epithelial cells as the latter present diminished COX-2 expression after treatment with TGF-β1. They may represent cells undergoing EMT in the fibrotic foci, or elsewhere in the interstitial space.

The mechanisms of COX-2 inhibition in the myofibroblast phenotype remain unknown. Recent findings suggest that defective histone acetylation in the COX-2 promoter may be responsible for the diminished COX-2 gene transcription observed in IPF fibroblasts [34]. Since PGE_2_ has shown potent anti-fibrotic properties in several studies [9]–[13], the effect of an altered secretion of PGE_2_ could easily be linked to the development of fibrotic processes. Further studies are needed to clarify whether the abrogation of COX-2 in the myofibroblasts of IPF is irreversible.

### Conclusions

The myofibroblast phenotype is associated with a down-regulation of COX-2 and, consequently, a reduced production of its main metabolite PGE_2_. These events could be crucial in IPF development and progression. Finally, our results suggest that targeting COX-2 and/or PGE_2_ could be a potential therapy for IPF.

## Supporting Information

Figure S1 **Expression of COX-2, COX-1 and α-SMA in control (n = 5) and IPF (n = 5) fibroblasts basally and induced by IL-1β (10 ng/ml**) **for 4 and 24 h.** (A) Representative image of a Western blot and (B) densitometric analysis of COX-2 expressed as ratio versus β-actin expression. *P<0.05 compared to respective untreated cells, #P<0.05 compared to control group in same conditions.
(TIF)

Figure S2 **Protein levels of COX-2, COX-1, α-SMA and β-actin in control and IPF fibroblasts stimulated for 4 h.** Cells were incubated in the presence or absence of IL-1β (10 ng/ml) and/or TGF-β1 (5 ng/ml) for 4****h. (A) Representative image of a Western blot. (B) Densitometric analysis of COX-2 expressed as ratio versus β-actin expression. * P<0.05 compared to respective untreated cells, † P<0.05 compared to IL-1β treated cells in the same group.
(TIF)

Figure S3 **Absence of COX-2 expression in control cells stimulated for 72 h with TGF-β1 and further incubation for 4 or 24 h with renewed TGF-β1.** Cells were incubated in the presence or absence of TGF-β1 (5 ng/ml) for 72 h and for additional 4 or 24 h of fresh TGF-β1 (5 ng/ml). Representative Western blot of a control fibroblast culture.
(TIF)

## References

[1] Hinz B, Phan SH, Thannickal VJ, Galli A, Bochaton-Piallat ML, et al (2007) The myofibroblast: one function, multiple origins. Am J Pathol 170: 1807–1816. 17525249 10.2353/ajpath.2007.070112 PMC1899462

[2] Willis BC, duBois RM, Borok Z (2006) Epithelial origin of myofibroblasts during fibrosis in the lung. Proc Am Thorac Soc 3: 377–382. 16738204 10.1513/pats.200601-004TK PMC2658689

[3] Broekelmann TJ, Limper AH, Colby TV, McDonald JA (1991) Transforming growth factor beta 1 is present at sites of extracellular matrix gene expression in human pulmonary fibrosis. Proc Natl Acad Sci U S A 88: 6642–6646. 1862087 10.1073/pnas.88.15.6642 PMC52144

[4] Uhal BD, Kim JK, Li X, Molina-Molina M (2007) Angiotensin-TGF-beta 1 crosstalk in human idiopathic pulmonary fibrosis: autocrine mechanisms in myofibroblasts and macrophages. Curr Pharm Des 13: 1247–1256. 17504233 10.2174/138161207780618885

[5] Brock TG, McNish RW, Peters-Golden M (1999) Arachidonic acid is preferentially metabolized by cyclooxygenase-2 to prostacyclin and prostaglandin E2. J Biol Chem 274: 11660–11666. 10206978 10.1074/jbc.274.17.11660

[6] Bonner JC, Rice AB, Ingram JL, Moomaw CR, Nyska A, et al (2002) Susceptibility of cyclooxygenase-2-deficient mice to pulmonary fibrogenesis. Am J Pathol 161: 459–470. 12163371 10.1016/S0002-9440(10)64202-2 PMC1850724

[7] Hodges RJ, Jenkins RG, Wheeler-Jones CP, Copeman DM, Bottoms SE, et al (2004) Severity of lung injury in cyclooxygenase-2-deficient mice is dependent on reduced prostaglandin E(2) production. Am J Pathol 165: 1663–1676. 15509536 10.1016/S0002-9440(10)63423-2 PMC1618657

[8] Horowitz JC, Peters-Golden M (2010) Prostaglandin E2's new trick: “decider” of differential alveolar cell life and death. Am J Respir Crit Care Med 182: 2–3. 20601589 10.1164/rccm.201002-0239ED

[9] Elias JA, Rossman MD, Zurier RB, Daniele RP (1985) Human alveolar macrophage inhibition of lung fibroblast growth. A prostaglandin-dependent process. Am Rev Respir Dis 131: 94–99. 3966717 10.1164/arrd.1985.131.1.94

[10] Saltzman LE, Moss J, Berg RA, Hom B, Crystal RG (1982) Modulation of collagen production by fibroblasts. Effects of chronic exposure to agonists that increase intracellular cyclic AMP. Biochem J 204: 25–30. 6288014 10.1042/bj2040025 PMC1158311

[11] Kohyama T, Ertl RF, Valenti V, Spurzem J, Kawamoto M, et al (2001) Prostaglandin E(2) inhibits fibroblast chemotaxis. Am J Physiol Lung Cell Mol Physiol 281: L1257–1263. 11597918 10.1152/ajplung.2001.281.5.L1257

[12] Kolodsick JE, Peters-Golden M, Larios J, Toews GB, Thannickal VJ, et al (2003) Prostaglandin E2 inhibits fibroblast to myofibroblast transition via E. prostanoid receptor 2 signaling and cyclic adenosine monophosphate elevation. Am J Respir Cell Mol Biol 29: 537–544. 12738687 10.1165/rcmb.2002-0243OC

[13] Huang SK, White ES, Wettlaufer SH, Grifka H, Hogaboam CM, et al (2009) Prostaglandin E(2) induces fibroblast apoptosis by modulating multiple survival pathways. FASEB J 23: 4317–4326. 19671668 10.1096/fj.08-128801 PMC2812040

[14] Borok Z, Gillissen A, Buhl R, Hoyt RF, Hubbard RC, et al (1991) Augmentation of functional prostaglandin E levels on the respiratory epithelial surface by aerosol administration of prostaglandin E. Am Rev Respir Dis. 144: 1080–1084. 10.1164/ajrccm/144.5.1080 1952435

[15] Wilborn J, Crofford LJ, Burdick MD, Kunkel SL, Strieter RM, et al (1995) Cultured lung fibroblasts isolated from patients with idiopathic pulmonary fibrosis have a diminished capacity to synthesize prostaglandin E2 and to express cyclooxygenase-2. J Clin Invest 95: 1861–1868. 7706493 10.1172/JCI117866 PMC295728

[16] Xaubet A, Roca-Ferrer J, Pujols L, Ramirez J, Mullol J, et al (2004) Cyclooxygenase-2 is up-regulated in lung parenchyma of chronic obstructive pulmonary disease and down-regulated in idiopathic pulmonary fibrosis. Sarcoidosis Vasc Diffuse Lung Dis 21: 35–42. 15127973

[17] Maher TM, Evans IC, Bottoms SE, Mercer PF, Thorley AJ, et al (2010) Diminished prostaglandin E2 contributes to the apoptosis paradox in idiopathic pulmonary fibrosis. Am J Respir Crit Care Med 182: 73–82. 20203246 10.1164/rccm.200905-0674OC PMC2902759

[18] American Thoracic Society (2000) Idiopathic pulmonary fibrosis: diagnosis and treatment. International consensus statement. American Thoracic Society (ATS), and the European Respiratory Society (ERS). Am J Respir Crit Care Med 161: 646–664. 10673212 10.1164/ajrccm.161.2.ats3-00

[19] Roca-Ferrer J, Garcia-Garcia FJ, Pereda J, Perez-Gonzalez M, Pujols L, et al (2011) Reduced expression of COXs and production of prostaglandin E(2) in patients with nasal polyps with or without aspirin-intolerant asthma. J Allergy Clin Immunol 128: 66–72. 21397936 10.1016/j.jaci.2011.01.065

[20] Ling H, Jia X, Zhang Y, Gapter LA, Lim YS, et al (2010) Pachymic Acid Inhibits Cell Growth and Modulates Arachidonic Acid Metabolism in Nonsmall Cell Lung Cancer A549 Cells. Mol Carcinog 49: 271–82. 19918789 10.1002/mc.20597

[21] Kuhn C, McDonald JA (1991) The roles of the myofibroblast in idiopathic pulmonary fibrosis. Ultrastructural and immunohistochemical features of sites of active extracellular matrix synthesis. Am J Pathol 138: 1257–1265. 2024710 PMC1886011

[22] Ramos C, Montaño M, García-Alvarez J, Ruiz V, Uhal BD, et al (2001) Fibroblasts from idiopathic pulmonary fibrosis and normal lungs differ in growth rate, apoptosis, and tissue inhibitor of metalloproteinases expression. Am J Respir Cell Mol Biol 24: 591–598. 11350829 10.1165/ajrcmb.24.5.4333

[23] Fang HY, Lin CY, Chow KC, Huang HC, Ko WJ (2010) Microarray detection of gene overexpression in primary spontaneous pneumothorax. Exp Lung Res 36: 323–330. 20653473 10.3109/01902141003628579

[24] McAnulty RJ, Chambers RC, Laurent GJ (1995) Regulation of fibroblast procollagen production. Transforming growth factor-beta 1 induces prostaglandin E2 but not procollagen synthesis via a pertussis toxin-sensitive G-protein. Biochem J 307: 63–68. 7717995 10.1042/bj3070063 PMC1136745

[25] Keerthisingam CB, Jenkins RG, Harrison NK, Hernandez-Rodriguez NA, Booth H, et al (2001) Cyclooxygenase-2 deficiency results in a loss of the anti-proliferative response to transforming growth factor-beta in human fibrotic lung fibroblasts and promotes bleomycin-induced pulmonary fibrosis in mice. Am J Pathol 158: 1411–22. 11290559 10.1016/s0002-9440(10)64092-8 PMC1891895

[26] Matsumura T, Suzuki T, Aizawa K, Sawaki D, Munemasa Y, et al (2009) Regulation of transforming growth factor-beta-dependent cyclooxygenase-2 expression in fibroblasts. J Biol Chem 18 284: 35861–71. 10.1074/jbc.M109.014639 PMC2791015 19837676

[27] Diaz A, Chepenik KP, Korn JH, Reginato AM, Jimenez SA (1998) Differential regulation of cyclooxygenases 1 and 2 by interleukin-1β, tumour necrosis factor-α and transforming growth factor-β1 in human lung fibroblasts. Exp Cell Res 241: 222–229. 9633531 10.1006/excr.1998.4050

[28] Kasai H, Allen JT, Mason RM, Kamimura T, Zhang Z (2005) TGF-beta1 induces human alveolar epithelial to mesenchymal cell transition (EMT). Respir Res 6: 56. 15946381 10.1186/1465-9921-6-56 PMC1177991

[29] Ramos C, Becerril C, Montano M, Garcia-De-Alba C, Ramirez R, et al (2010) FGF-1 reverts epithelial-mesenchymal transition induced by TGF-{beta}1 through MAPK/ERK kinase pathway. Am J Physiol Lung Cell Mol Physiol 299: L222–231. 20495078 10.1152/ajplung.00070.2010

[30] Mitchell JA, Belvisi MG, Akarasereenont P, Robbins RA, Kwon OJ, et al (1994) Induction of cyclo-oxygenase-2 by cytokines in human pulmonary epithelial cells: regulation by dexamethasone. Br J Pharmacol 113: 1008–1014. 7858842 10.1111/j.1476-5381.1994.tb17093.x PMC1510466

[31] Anscher MS (2010) Targeting the TGF-beta1 pathway to prevent normal tissue injury after cancer therapy. Oncologist 15: 350–359. 20413640 10.1634/theoncologist.2009-S101 PMC3227962

[32] Singh SR, Hall IP (2008) Airway myofibroblasts and their relationship with airway myocytes and fibroblasts. Proc Am Thorac Soc 5: 127–132. 18094095 10.1513/pats.200706-070VS

[33] Lappi-Blanco E, Kaarteenaho-Wiik R, Maasilta PK, Anttila S, Paakko P, et al (2006) COX-2 is widely expressed in metaplastic epithelium in pulmonary fibrous disorders. Am J Clin Pathol 126: 717–724. 17050069 10.1309/PFGX-CLNG-2N17-PJX9

[34] Coward WR, Watts K, Feghali-Bostwick CA, Knox A, Pang L (2009) Defective histone acetylation is responsible for the diminished expression of cyclooxygenase 2 in idiopathic pulmonary fibrosis. Mol Cell Biol 29: 4325–4339. 19487460 10.1128/MCB.01776-08 PMC2715818

