# Supplementary figures and images for: Lung Myofibroblasts Are Characterized by Down-Regulated Cyclooxygenase-2 and Its Main Metabolite, Prostaglandin E2
Source: PLoS One. 2013 Jun 3;8(6):e65445. doi: 10.1371/journal.pone.0065445 (PMC3670886; doi:10.1371/journal.pone.0065445)

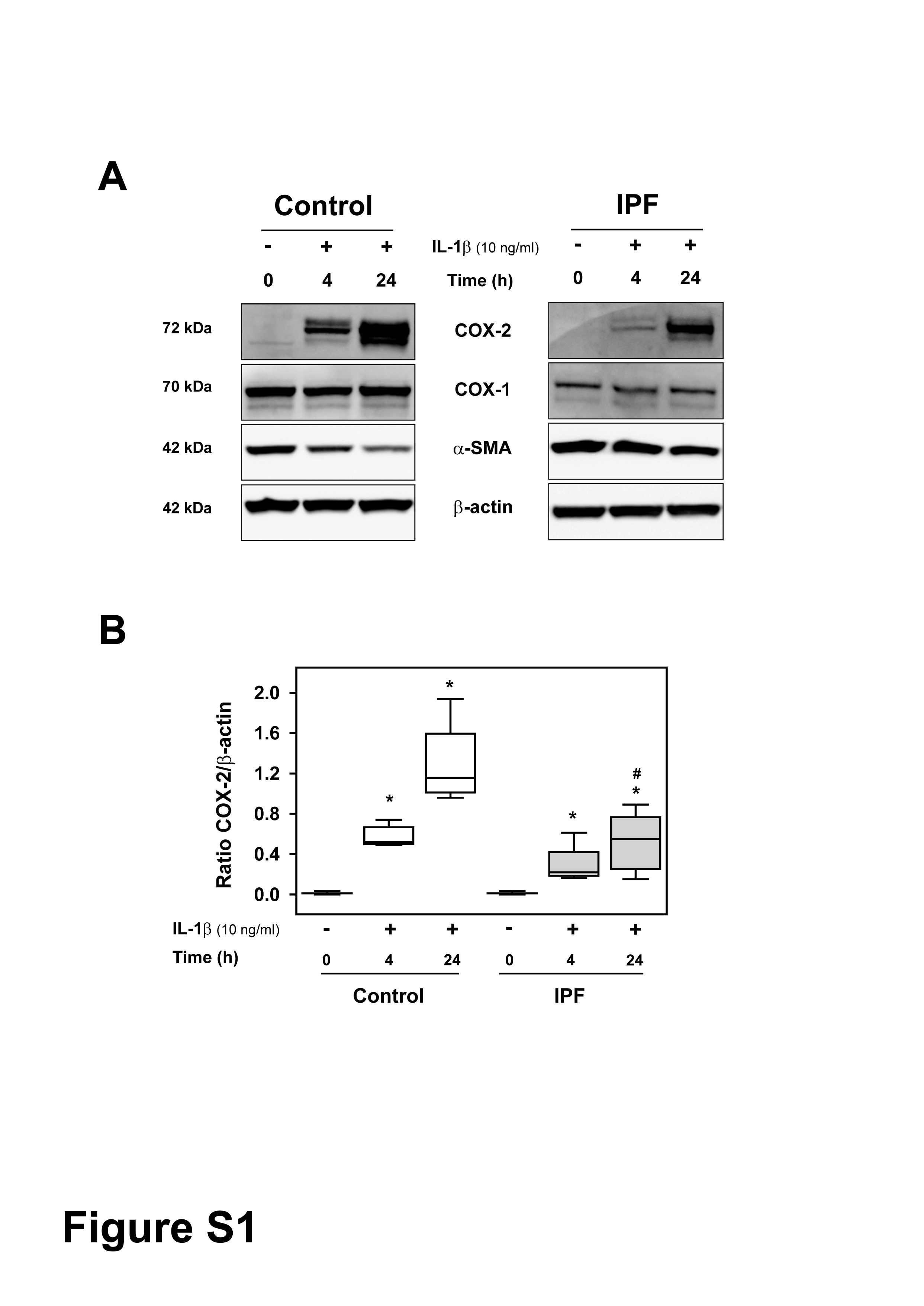

Supplement: Figure S1 — Expression of COX-2, COX-1 and α-SMA in control (n = 5) and IPF (n = 5) fibroblasts basally and induced by IL-1β (10 ng/ml) for 4 and 24 h. (A) Representative image of a Western blot and (B) densitometric analysis of COX-2 expressed as ratio versus β-actin expression. *P<0.05 compared to respective untreated cells, #P<0.05 compared to control group in same conditions. (TIF) [file pone.0065445.s001.tif]

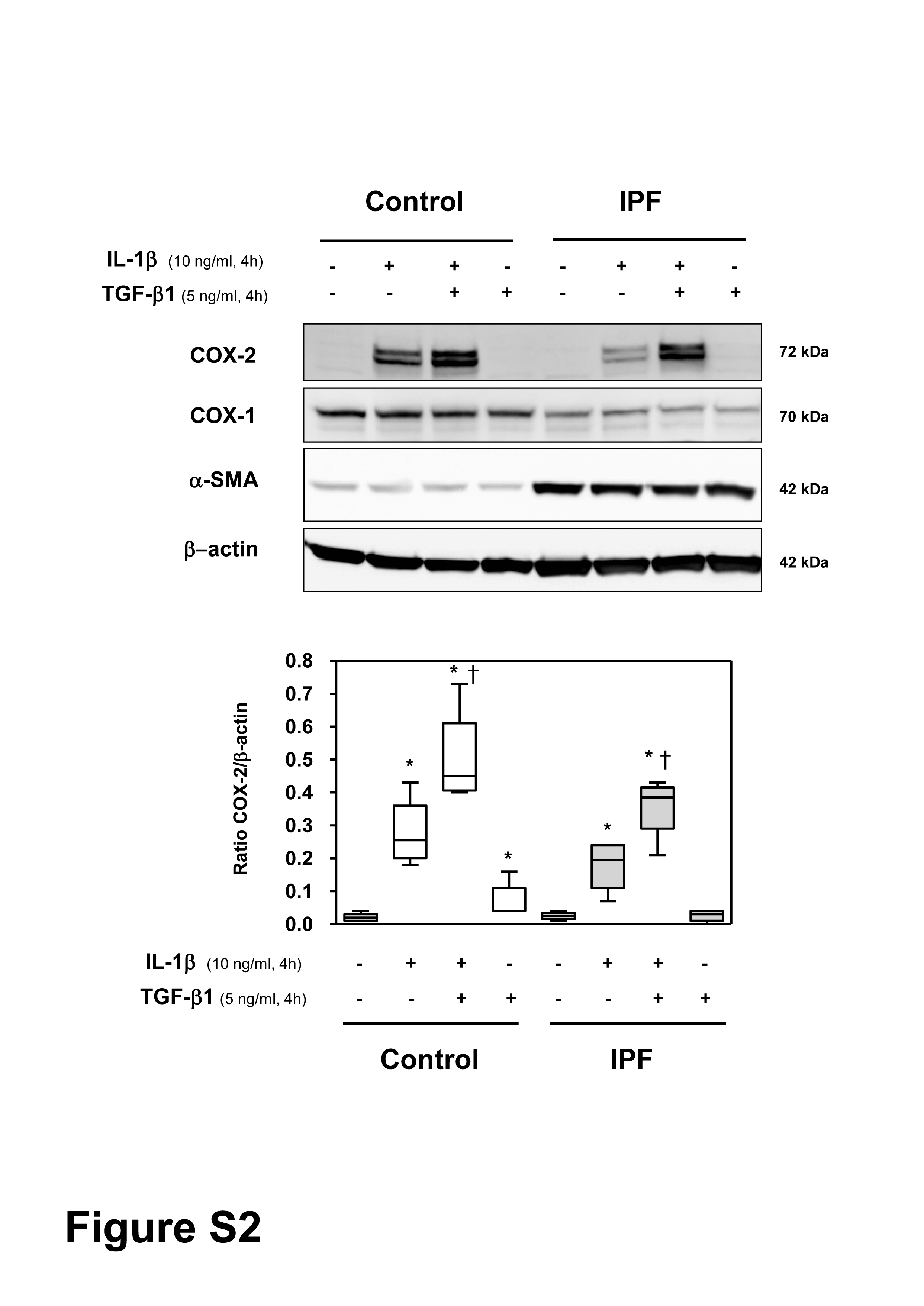

Supplement: Figure S2 — Protein levels of COX-2, COX-1, α-SMA and β-actin in control and IPF fibroblasts stimulated for 4 h. Cells were incubated in the presence or absence of IL-1β (10 ng/ml) and/or TGF-β1 (5 ng/ml) for 4h. (A) Representative image of a Western blot. (B) Densitometric analysis of COX-2 expressed as ratio versus β-actin expression. * P<0.05 compared to respective untreated cells, † P<0.05 compared to IL-1β treated cells in the same group. (TIF) [file pone.0065445.s002.tif]

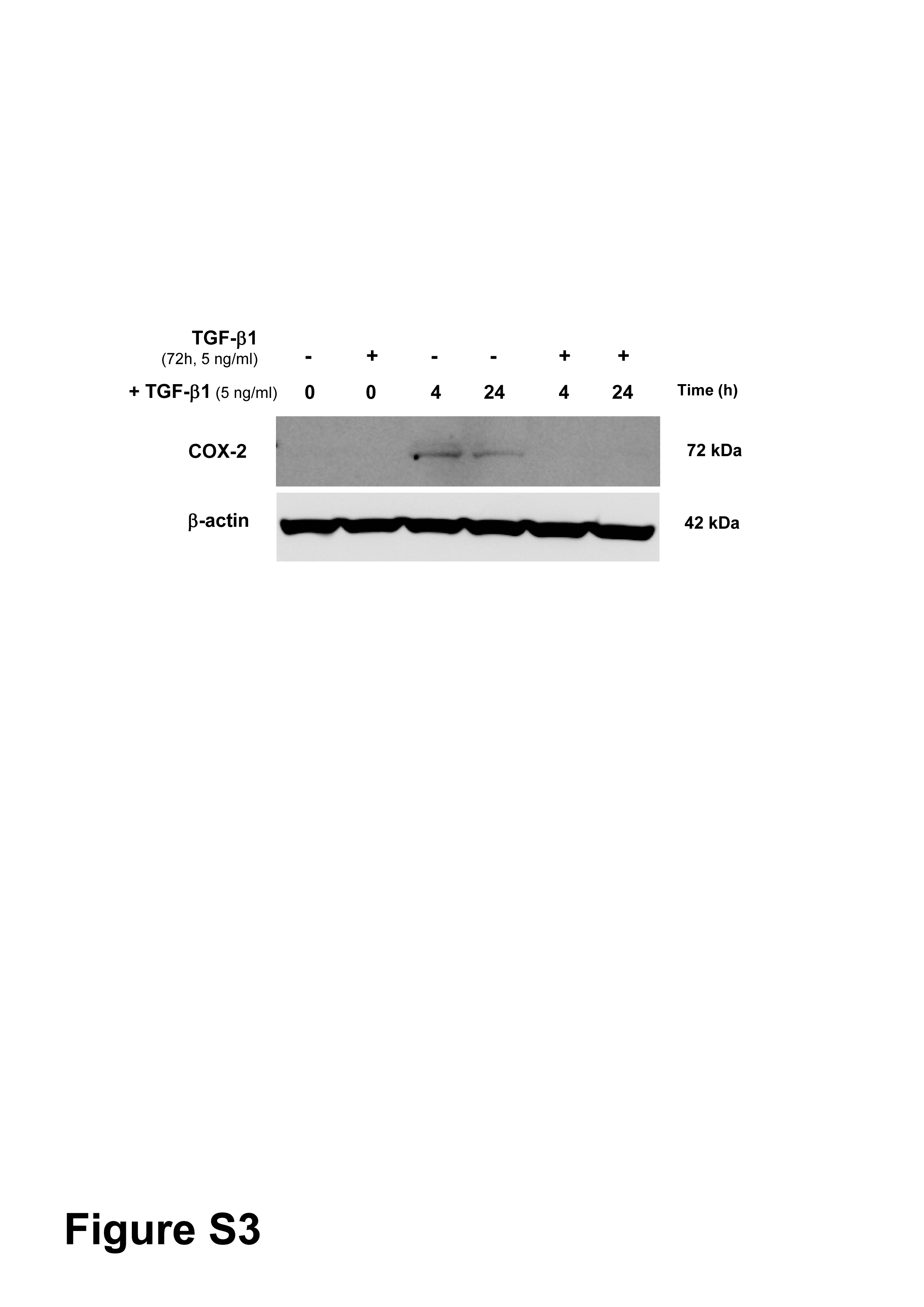

Supplement: Figure S3 — Absence of COX-2 expression in control cells stimulated for 72 h with TGF-β1 and further incubation for 4 or 24 h with renewed TGF-β1. Cells were incubated in the presence or absence of TGF-β1 (5 ng/ml) for 72 h and for additional 4 or 24 h of fresh TGF-β1 (5 ng/ml). Representative Western blot of a control fibroblast culture. (TIF) [file pone.0065445.s003.tif]
